# A combined-method approach to trace submarine groundwater discharge from a coastal karst aquifer in Ireland

**DOI:** 10.1007/s10040-019-02082-0

**Published:** 2019-12-09

**Authors:** Philip Schuler, L. Stoeckl, P.-A. Schnegg, C. Bunce, L. Gill

**Affiliations:** 1 Department of Civil, Structural and Environmental Engineering, University of Dublin Trinity College, Dublin 2, Ireland; 2 Department for Groundwater Resources – Quality and Dynamics, Federal Institute for Geosciences and Natural Resources (BGR), Stilleweg 2, 30655 Hannover, Germany; 3 Albillia Co., CH2000 Neuchâtel, Switzerland; 4 Burren Outdoor and Education Centre (BOEC), Turlough, Clare Ireland

**Keywords:** Coastal aquifer, Tracer test, Submarine groundwater discharge, Remote sensing, Ireland

## Abstract

Knowledge about the hydraulic connections between submarine groundwater discharge (SGD) and its terrestrial coastal catchment is relevant with regard to the management of marine and coastal waters in karst areas. This study applies different methods and monitoring approaches to trace SGD between the Burren Limestone Plateau and Galway Bay in western Ireland, via an excavated sinkhole shaft and deep conduit. Areas of potential SGD were first delineated based on sea surface temperature anomalies using Landsat satellite images. Two fluorescent dyes and solid wood chips were then used as tracers. Solid wood chips were tested as potential means to circumvent the problem of high dispersion in the sea, impacting on the fluorescent dyes to yield readings below the detection limits. Sampling was conducted at 10 different terrestrial locations and in the sea at Galway Bay. Offshore sampling was conducted in transects over a period of four successive days onboard of a vessel using an automated field fluorometer and a conductivity-temperature-depth sensor. No wood chips were recovered in the sea but both fluorescent dyes were successfully sampled. The estimated travel times are in the order of 100 to 354 m/h, and localised tracer readings correlate well in space and time with low conductivity readings. By confirming hydraulic connections between the two karst features and Galway Bay, the study substantiates the hypothesised importance of Variscan veins with regard to regional groundwater flow in the region.

## Zusammenfassung

Die Kenntnis hydraulischer Zusammenhänge zwischen untermeerischen Grundwasseraustritten und deren terrestrischen Küsteneinzugsgebieten ist für die Bewirtschaftung der Meeres- und Küstengewässer in Karstgebieten von Bedeutung. Diese Studie wendet verschiedene Methoden und Messansätze an, um untermeerische Grundwasseraustritte zwischen dem Burren Kalkstein Plateau und der Galway Bucht in Westirland über einen ausgegrabenen Dolinen-Schacht und eine tiefe Karströhre zu verfolgen. Meeresbereiche mit potenziellen Grundwasseraustritten wurden zuerst unter Verwendung von Landsat-Satellitenbildern auf Grundlage von Temperaturanomalien an der Meeresoberfläche abgegrenzt. Dann wurden zwei fluoreszierende Farbstoffe und Holzspäne als Tracer verwendet. Die Holzspäne wurden als Mittel zur Umgehung des Problems starker Dispersion im Meer getestet, welche sich potentiell auf die fluoreszierenden Farbstoffe auswirken, und in Messwerten unterhalb der Nachweisgrenze resultieren kann. Die Probenahme wurde an 10 verschiedenen terrestrischen Standorten und im Meer der Galway Bucht durchgeführt. Mit einem automatisierten Feldfluorometer und einer Leitfähigkeits-, Temperatur- und Tiefensonde wurden in den jeweiligen Meeressektoren Messungen über einen Zeitraum von vier aufeinanderfolgenden Tagen an Bord eines Schiffes durchgeführt. Zwar wurden im Meer keine Holzspäne zurückgewonnen, allerdings wurden beide Fluoreszenzfarbstoffe erfolgreich gemessen. Die geschätzten Fließzeiten liegen in der Größenordnung von 100 bis 354 m/h und lokalisierte Tracer-Messwerte korrelieren räumlich und zeitlich gut mit Messwerten geringerer Leitfähigkeit. Die Bestätigung der hydraulischen Verbindungen zwischen dem Kalksteinplateau und der Bucht von Galway bestärkt die Hypothese der Bedeutsamkeit von variszischen Adern für den regionalen Grundwasserfluss.

## Résumé

La connaissance des connexions hydrauliques entre les zones de sorties d’eaux souterraines sous-marines et le bassin terrestre côtier attenant est pertinente pour la gestion des eaux marines et côtières en zones karstiques. Cette étude applique différentes approches méthodologiques et de suivi pour tracer la sortie d’eaux souterraines sous-marines entre le plateau calcaire de Burren et la baie de Galway en Irlande de l’Ouest, via une perte et un conduit profond. Les zones de sorties potentielles d’eaux souterraines sous-marines ont été délimitées dans un premier temps sur la base d’anomalies de température des eaux de mer en utilisant des images satellites Landsat. Deux traceurs fluorescents et des copeaux de bois ont été utilisés comme traceurs. Les copeaux de bois ont été utilisés comme moyen potentiel pour contourner les problèmes de dispersion élevée dans la mer, qui impactent les traceurs fluorescents et engendrent des concentrations sous les limites de détections. L’échantillonnage a été menée sur dix sites différents à terre et en mer dans la baie de Galway. L’échantillonnage en mer e a été mené selon des profils sur une période de quatre jours successifs à bord d’un navire muni d’un fluorimètre de terrain automatique et d’un capteur de conductivité-température-profondeur. Aucun copeau de bois n’a été récupéré en mer, mais les deux traceurs fluorescents ont été échantillonnés avec succès. Les temps de séjour estimés sont de l’ordre de 100 à 354 m/h, et les valeurs de traceurs localisées corrèlent bien en espace et en temps avec les basses valeurs de conductivité électrique. En confirmant les connexions hydrauliques entre les deux phénomènes karstiques et la baie de Galway, l’étude corrobore l’hypothèse de l’importance des veines du cycle varisque sur l’organisation des écoulements régionaux d’eau souterraine dans la région.

## Resumen

El conocimiento de las conexiones hidráulicas entre la descarga submarina de aguas subterráneas (SGD) y su cuenca costera terrestre es relevante para la gestión de las aguas marinas y costeras en las zonas kársticas. Este estudio aplica diferentes métodos y enfoques de monitoreo para el seguimiento de la SGD entre la meseta de caliza de Burren y la bahía de Galway en el oeste de Irlanda, a través de un pozo excavado y un conducto profundo. Las áreas de SGD potenciales fueron delineadas primero basándose en anomalías de la temperatura de la superficie del mar usando imágenes satelitales Landsat. Dos tintes fluorescentes y astillas de madera maciza se utilizaron como trazadores. Las astillas de madera maciza se probaron como medio potencial para eludir el problema de la alta dispersión en el mar, impactando en los colorantes fluorescentes para obtener lecturas por debajo de los límites de detección. El muestreo se llevó a cabo en 10 lugares terrestres diferentes y en el mar en la Bahía de Galway. El muestreo en alta mar se llevó a cabo en transectas durante un período de cuatro días consecutivos a bordo de un buque utilizando un fluorómetro de campo automatizado y un sensor de conductividad-temperatura-profundidad. No se recuperaron astillas de madera en el mar, pero se tomaron muestras exitosas de ambos tintes fluorescentes. Los tiempos de tránsito estimados son del orden de 100 a 354 m/h, y las lecturas del trazador localizadas se correlacionan bien en el espacio y el tiempo con las lecturas de baja conductividad. Al confirmar las conexiones hidráulicas entre las dos características kársticas y la Bahía de Galway, el estudio corrobora la importancia hipotética de las vetas de Variscan con respecto al flujo regional de agua subterránea en la región.

## 摘要

在岩溶地区, 海底地下水排泄(SGD)与其陆地沿海集水区之间水力联系的认识与海洋和沿海水域的管理有关。本研究采用不同的方式和监测方法, 通过开挖的落水洞和深水管道来示踪爱尔兰西部Burren石灰岩高原和Galway湾之间的地下水排泄。首先利用Landsat卫星图像和基于海平面温度异常划定了潜在的SGD区域。然后将两种荧光染料和实木碎片用作示踪剂。测试了实木碎屑作为解决海洋中由于影响荧光染料使产生低于检测极限读数的高弥散问题的潜在手段。在Galway湾的10个不同陆面位置和海洋中进行了采样。使用自动现场荧光计和电导率-温度-深度传感器, 在船上连续四天的时间段内, 进行海洋取样。在海洋中没有回收木屑, 但两种荧光染料均已成功采样。估计的行程时间约为100至354 m/h, 局部示踪剂读数在空间和时间上与低电导率读数相关性很好。通过确定两个喀斯特地貌与Galway湾之间的水力联系, 该研究证实了华力西期脉对于该地区区域地下水流动的假设重要性。

## Resumo

O conhecimento sobre as conexões hidráulicas entre a descarga submarina de águas subterrâneas (DSAS) e sua captação terrestre na costa é relevante no que diz respeito ao gerenciamento de águas marinhas e costeiras em áreas cársticas. Este estudo aplica diferentes métodos e abordagens de monitoramento para rastrear a DSAS entre o platô de calcário de Burren e a baía de Galway, no oeste da Irlanda, por meio de um poço escavado e um conduto profundo. Áreas de potencial DSAS foram primeiramente delineadas com base em anomalias de temperatura da superfície do mar usando imagens do satélite Landsat. Dois corantes fluorescentes e lascas de madeira maciça foram então utilizados como marcadores. Lascas de madeira maciça foram testadas como um meio potencial para contornar o problema de alta dispersão no mar, impactando nos corantes fluorescentes para produzir leituras abaixo dos limites de detecção. A amostragem foi realizada em 10 locais terrestres diferentes e no mar na Baía de Galway. A amostragem fora da costa foi realizada em transectos durante um período de quatro dias sucessivos a bordo de uma embarcação usando um fluorômetro de campo automatizado e um sensor de condutividade-temperatura-profundidade. Nenhuma lasca de madeira foi recuperada no mar, mas os dois corantes fluorescentes foram amostrados com sucesso. Os tempos de viagem estimados são da ordem de 100 a 354 m/h, e as leituras localizadas do traçador se correlacionam bem no espaço e no tempo com as leituras de baixa condutividade. Ao confirmar as conexões hidráulicas entre as duas formações cársticas e a Baía de Galway, o estudo confirma a hipótese da importância das veias variscas em relação ao fluxo regional de águas subterrâneas na região.

## Introduction

Artificial tracer tests are common methods in karst hydrogeology (Kaess 1998; Benischke et al. 2007) to study conduit parameters (Geyer et al. 2007; Luhmann et al. 2012), the transportation characteristics of potential contaminants (Flynn and Sinreich 2010), or more generally to establish hydraulic connections and estimate transit times (Lauber and Goldscheider 2014; Margane et al. 2018). Fluorescent dyes are commonly used such as uranine (sodium fluorescein), rhodamines or optical brightener, as are physico/chemical tracers such as chloride and temperature (Luhmann et al. 2012) or particulate bacteriophages (Sinreich and Flynn 2006; Maurice et al. 2010). Artificial tracers are usually applied between a defined injection site (e.g. sinkhole, underground river, or borehole) and a discrete sampling site, usually a spring. Tracer studies are most commonly executed within terrestrial catchments.

Many karst catchments are coastal, discharging via submarine springs. Eustatic sea level variations down to −120 m below modern sea level result in a range of lower base levels in coastal aquifers globally. Off the Republic of Ireland, sea levels are estimated to have dropped by 60–100 m below the present level at the Irish coast and Galway Bay between 15 and 26 ka ago (Edwards and Craven 2017; O’Connell and Molloy 2017). As a result, karst morphology extends beyond the shore into the sea, as is the case for the Burren Plateau (Kozich and Sautter 2018).

Submarine and intertidal groundwater discharge (SiGD) or purely submarine groundwater discharge (SGD) has received increased attention over the recent decades, and these fluxes are internationally recognized as an important pathway for transport into the coastal environment, particularly in karst areas (Taniguchi et al. 2002; Burnett et al. 2006; Dimova et al. 2011; Montiel et al. 2018). Knowledge about the occurrence and quantification of SiGD/SGD is mostly relevant in terms of understanding the hydraulics and hydrology of catchments (Smith and Nield 2003; Peterson et al. 2008) and/or to estimate nutrient fluxes from the land into the sea (Santos et al. 2008; Lee et al. 2012; McCormack et al. 2014; Null et al. 2014), potentially causing adverse impacts such as algal blooms (Silke et al. 2005; Green et al. 2014; Li et al. 2017) together with negative impacts on the ecology or the mariculture industry (Laroche et al. 1997). Equally, such fluxes may attract specific ecology, for example, the occurrence of the endangered species undulate ray (*Raja undulata*) is associated with freshwater inputs into the sea, including along the Irish coast (Ellis et al. 2012); species protection therefore can also be linked to onshore catchment dynamics.

Locating diffuse or point SGD and further determining the associated terrestrial catchment is a challenge in itself due to the inherent significant spatial and temporal variability in the flux (Burnett and Dulaiova 2003). Natural environmental tracers such as salinity, temperature or radon may be sampled in situ (Schubert et al. 2014), while some tracers, like temperature, may be also remotely sensed from space (Zektser et al. 2007; Johnson et al. 2008; Wilson and Rocha 2012; Tamborski et al. 2015). In all cases, the use of such parameters is constrained by ocean mixing and dilution, thereby weakening and spatially integrating the signal potential towards a limit of detection or below (Breier et al. 2005).

Within the context of karst, abundant studies exist relating to: (1) the detection and/or quantification of SGD, and (2) the use of artificial tracer methods for onshore catchment hydrogeology. Yet, until present, to the knowledge of the authors, there is no systematic approach which links the study of SGD with artificial tracer tests, despite the prevalence of tracer studies in onshore catchment hydrogeology (Benischke et al. 2007), and the clear need to link the SGD to its catchment. This research uses a combined-method approach to: (1) locate offshore areas of SGD using remote sensing, and (2) use different artificial and natural tracers to evaluate hydraulic connections between terrestrial injection points and marine SGD locations. More specifically, this study presents an approach for tracing SGD to onshore locations of a coastal telogenetic karst aquifer in combination with remote sensing techniques. Such knowledge can then be used to more effectively link the management of land use with coastal water quality.

## Materials and methods

### Study area

#### Geography, geology, structure

**Fig. 1:**
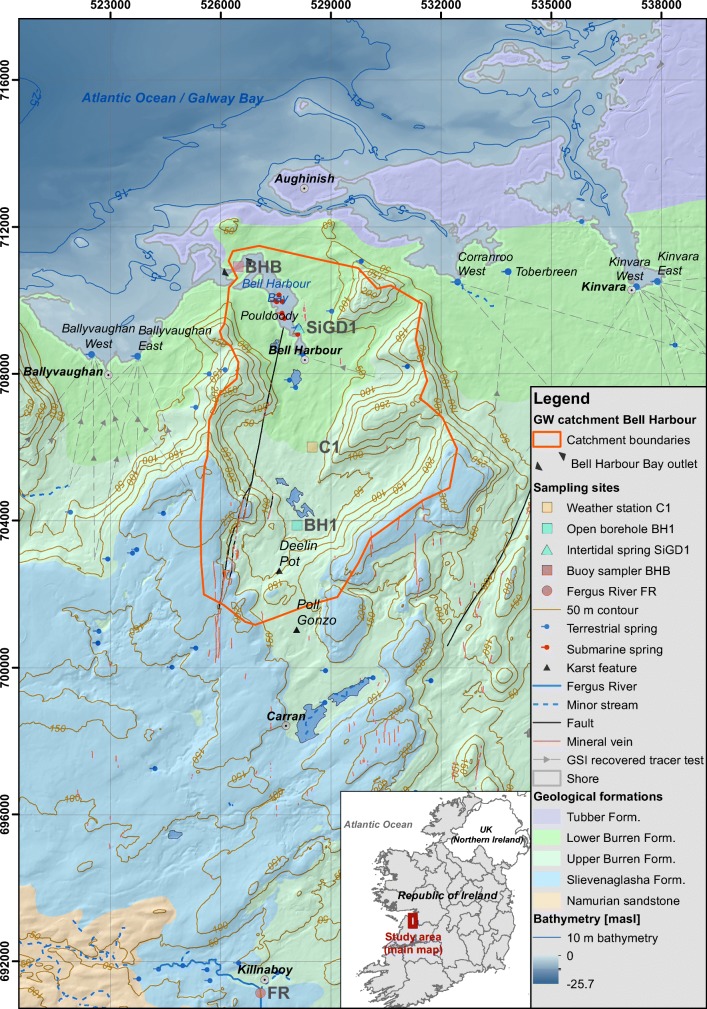
Site area of Bell Harbour in the west of Ireland, County Clare: topography, geology (MacDermot et al. 2003), structure, terrestrial (GSI 1999) and submarine (O’Connell et al. 2018) springs, successful groundwater traces (GSI 2017), and sampling locations of the project used in this study

The groundwater catchment of Bell Harbour (Fig. 1)—as previously adopted by McCormack et al. (2017); Schuler et al. (2018)—is located in the north-eastern part of the Burren Limestone Plateau in the west of the Republic of Ireland. The Burren, including the upland catchment of Bell Harbour, is a temperate glaciokarst landscape, which has been subject to repeated glaciation during the Pleistocene, showing features typical of glaciation such as ice-plucked crags, scoured rock surfaces, limestone pavements and erratic boulders (Simms 2014). The mean annual air temperature is 13.6 °C and the annual rainfall was 1,386 and 1,560 mm for the hydrological years 2017 and 2018, respectively, as measured at the weather station C1 (Fig. 1). Multiple valleys intersect the study area that range in elevation between sea level in the north and up to 340 m above sea level (masl) along the escarpments. Along the escarpments the bare outcrop shows high degrees of karstification.

The entire area is underlain by Lower Carboniferous well-bedded and pure limestones ranging between the Tubber formation (early Viséan), the Lower Burren and Upper Burren formation (mid-Viséan, Asbian), and the Slievenaglasha Formation (late Viséan, Brigantian). The presence of irregular limestone surfaces is interpreted as palaeokarst (Pracht et al. 2015). The total thickness of limestone reaches at least 510 m. The strata dip generally to the south, ranging between 2 and 3°.

The deformation of Carboniferous rocks in Ireland is largely attributed to the Variscan orogeny and the associated north/northwest-oriented compression (Graham 2009). The Variscan contractional deformation caused the formation of calcite veins of a few microns to 0.5 m width, which are laterally and vertically consistent across bedding discontinuities (Gillespie et al. 2001).

A suite of veins with more exotic mineralisation, including fluorite, quartz and occasional sulphides, occurs in a ~10-km wide north–south zone centred near Carran (see Fig. 1). These generally wider veins range from 0.05 to 1 m, and are part of a structure which extends 20 km across all stratigraphic sequences of the Burren (J. Walsh et al., University College Dublin, unpublished paper, 2019). The veins are visible in outcrop, and have been observed at more than 100 m depth, for example along the vertically oriented cave Poll Gonzo (Bunce 2010). The horizontal persistency of veins along strikes spans over 7 km, interconnecting caves (Mac Sharry 2006). Another example is the recently discovered sinkhole Deelin Pot, which is a 18 m deep and 5-m-wide excavated shaft oriented along north–south oriented calcite veins containing also silica veining, previously filled with glacial debris (Bunce and Drew 2017). Furthermore, slickenside veins were recorded at several depths in a 490-m deep borehole drilled by the Geological Survey Ireland (GSI) at location BH1 (Fig. 1).

The veins may be dissolved out or still intact. Where intact, the contact between limestone host rock and the (calcite) vein material may act as an inception horizon (Lowe and Waters 2014) and pathway of preferential dissolution of the limestone rock. The presence of sulphide minerals infill provides a source of sulphur for the formation of sulphuric acids to increase the rate of dissolution. Therefore, veins must be considered important for regional groundwater flow, as their lateral and vertical extent is believed to have largely contributed to north-south groundwater flow paths (Moore and Walsh 2013) and (J. Walsh et al., University College Dublin, unpublished paper, 2019). The joints pattern is very prominent, and it is known to extend several kilometres off-shore, at least to 80 m below sea level (mbsl; Kozich and Sautter 2018).

Offshore sinkholes are known to exist within Bell Harbour Bay. The use of marine electrical resistivity tomography (ERT) confirmed that these sinkholes are partly hydraulically activated, hence, acting as submarine springs. These sinkholes are generally filled with sediments, yet, there are single examples that indicate the absence of a sediment infill (O’Connell et al. 2018). The geology and structure of the telogenetic karst Burren Plateau extends several kilometres off-shore west of the Burren (Gillespie and Sautter 2018), and therefore sinkholes may accompany these structures, yet, to the knowledge of the authors, no detailed studies have yet confirmed this.

#### Groundwater flow and previous tracer tests

Approximately 60% of the Burren Plateau drains directly to the sea, and significant quantities of groundwater discharge occurs along the shore via intertidal springs (Drew 2018). The intertidal springs that are relevant to this study were previously traced successfully (Table 1) and are illustrated in Fig. 1. All tracer tests were carried out qualitatively; hence, the average flow velocities are approximated and range between 21 and 460 m/h. For the Bell Harbour catchment, only one successful tracer test was carried out over a distance of 2.2 km.

**Table 1:** Summary of successful tracer tests previously carried out to investigate intertidal groundwater discharge; source of data: Cronin et al. (1999) and GSI ( 2017). Note that fluorescent dyes were sampled qualitatively using activated charcoal for uranine and rhodamine WT, and cotton wool for leucophor

Intertidal spring	No. of tests	Date/year	Tracer used	Range of flow velocities
Ballyvaughan West	5	1999	Leucophor, Rhodamine WT, Uranine	21–150 m/h
Ballyvaughan East	5	1999	Leucophor, Rhodamine WT, Uranine	21–150 m/h
Bell Harbour	1	2003	Uranine	–
Corranroo West	2	21 Nov 1996	Bacteriophages (Psf2 and H40)	111–126 m/h
Toberbreen	1	1 Oct 1996	Leucophor	13–26 m/h
Kinvara West and Central	6	1 and 18 Oct 1996; 21 Nov 1996;	Bacteriophages (H4, H40, T7), Leucophor, Rhodamine WT, Uranine	47–460 m/h
Kinvara East	5	Jul, Aug and Sep 1990; 18 Oct 1996; 21 Nov 1996; 4 Dec 1996	Bacteriophages (Psf2), Leucophor, Rhodamine WT	150–460 m/h

Known locations of SGD are either in conjunction with intertidal springs along the western shore of the Burren Plateau or limited to Bell Harbour and Kinvara Bay (Drew 2003; O’Connell et al. 2018) with estimated discharge rates ranging between 0 and 4.3 m^3^/s, and 5–16 m^3^/s respectively (McCormack et al. 2014; Schuler et al. 2018). On a larger scale, significant areas of sea-surface-temperature anomalies interpreted as SGD were detected on the western side of the Burren Plateau linked to structural geology (Wilson and Rocha 2012). Due to the scale of these temperature anomalies, the rate of SGD may exceed the abovementioned numbers.

Within the Bell Harbour catchment, the groundwater flow is a conduit-dominated updip from south to north (McCormack et al. 2017). Discharge of the study area occurs via SiGD, including the intertidal spring Pouldoody, into Bell Harbour Bay. A shallow north–south conduit of ~2 m diameter is argued to link shallow flow within the catchment and springs in Bell Harbour Bay (McCormack et al. 2017; Fig. 1). The SiGD regime fluctuates seasonally corresponding to the overall piezometric state of the aquifer driving an overflow mechanism. Previous tracer tests suggest that groundwater flow velocities may reach up to 460 m/h (Table 1). Vertical groundwater flow was measured up to 176 mbsl in BH1 reaching up to 97 m/h. This deep flow was hypothesised to drain the coastal aquifer as SGD into Galway Bay as an additional outlet to the SiGD into Bell Harbour Bay (Schuler et al. 2018), with one goal of this study to provide confirmation by artificial tracer tests.

The high degree of karstification limits surface-water features to short reaches of ephemeral streams (Drew 1990). South of the study area and the Burren Plateau, the Fergus River drains large areas from the southern part of the Burren. Recharge in the study area originates from rainfall, percolating rapidly towards the phreatic zone based on the response of borehole hydrographs (Schuler et al. 2018). Recharge is assumed to get concentrated along preferential flow paths, as for example within Poll Gonzo, where an underground stream rapidly falls 40 m until reaching the permanent local water table at ~31 masl (Bunce 2010). Two tracer studies were conducted, in 2011 and 2015, using 5 kg of uranine and 4 kg of rhodamine WT, respectively, to trace flow paths from Poll Gonzo, but without success. In addition to the tracer tests illustrated in Fig. 1, 11 tracer tests have been carried out in the uplands of the Burren Plateau. Four of these tests investigated a hydraulic connection to the shore, yet, none proved confirmatory (Bunce and Drew 2017). Relatively fast, concentrated and mainly deep groundwater flow may have bypassed any tracer from shallow sampling locations. Furthermore, it is assumed that large dissolution features such as Deelin Pot must be well connected to such deep conduit network.

#### Hydroclimatic sampling and data

Within the study area, different sampling sites were set up to record hydroclimatic data. A full weather station at C1 (38 masl, Fig. 1) records rainfall in 15-min intervals using a CEL tipping-bucket rain gauge (Casella, Bedford, UK) attached to a Rainlogger Model 3002 (Solinst Canada Ltd., Ontario, Canada) data logger, wind speed in 15-min intervals using an ultrasonic wind sensor (Gill Instruments Ltd) attached to a CR800 data logger (Campbell Scientific Ltd., Shepshed, UK), and atmospheric pressure in 1-h intervals using an INW PT2X sensor (Seametrics, Kent, WA, USA). The water depth of the Fergus River at FR (~23 masl, Fig. 1) was monitored at 1-h intervals using a Mini-Diver502 (SchlumbergerWater Services, British Colombia, Canada). A stage-discharge curve was established through 13 spot discharge measurements using an OTT acoustic-digital-current meter (OTT Hydromet GmbH, Kempten, Germany). Temperature, level and electrical conductivity (temperature compensated) of intertidal groundwater discharge at Pouldoody spring at SiGD1 (−0.1 masl, Fig. 1) were measured in 1-h intervals using a Solinst LTC sensor (Solinst Canada Ltd., Canada). The LTC sampler at SiGD was fixed on a concrete platform placed into the near-shore sediments 1-2 m away and ~1 m below the spring outlet. A second Solinst LTC was used to monitor temperature, level and electrical conductivity (temperature compensated) in the sea onboard of a vessel. Bathymetry data used in this study comprise a 5- and 10-m grid provided by the GSI. Land topography is based on the Shuttle Radar Topography Mission (SRTM; USGS 2014).

### Remote sensing

Satellite imagery was used to identify offshore sampling areas based on expected temperature differences between the sea and emerging SGD (Roxburgh 1985; Schubert et al. 2014). Water surface temperature anomalies were identified using Landsat-8 Operational Land Imager (OLI) and Thermal Infrared Sensor (TIRS) images with <20% cloud cover from the US Geological Survey. Landsat-8 is an optical sensor with a spatial resolution of 15 m (band 8), 30 m (band 1–7 and 9) and 100 m (band 10–11, TIRS).

**Fig. 2:**
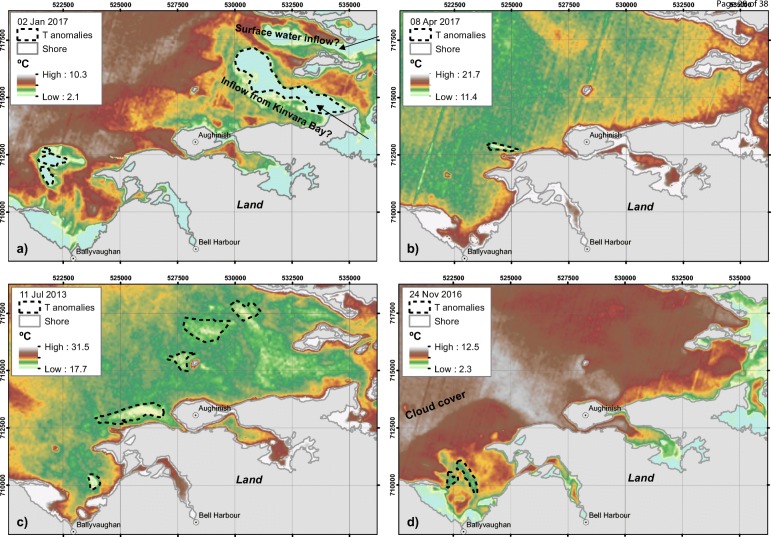
Sea surface temperatures derived from Landsat 8 OLI, indicating localised temperature anomalies indicative for submarine groundwater discharge (SGD) ordered by month of the year on **a** 2 January 2017, **b** 8 April 2017, **c** 11 July 2013 and **d** 24 November 2016. Note that temperature is scaled differently in each scene

Surface temperature grids in °C were generated in three steps in ArcMap (version 10.1, ESRI, Redlands, USA) using a toolbox for automated mapping (Walawender et al. 2012): (1) conversion of the thermal infrared band into brightness temperature (temperature of a black body in thermal equilibrium with its surroundings); (2) computation of the land surface emissivity via normalised differenced vegetation index (NDVI) using the red band and the near infrared band; and (3) calculation of the land surface temperature using the previous outputs along with atmospheric correction parameters to account for atmospheric transmissivity, up-welling atmospheric radiance, and down-welling atmospheric radiance. Atmospheric correction parameters were generated using the web-based Atmospheric Correction Parameter Calculator (Barsi et al. 2003). Four Landsat images were chosen as most suitable to estimate the sea surface temperatures dating 2 January 2017, 8 April 2017, 11 July 2013 and 24 November 2016. Only Fig. 2b included any visible distortion by cloud cover over the water surface. Temperature grids were visualised in ArcMap using multi-colour stretched patterns to facilitate the visual identification of localised temperature anomalies.

### Artificial tracer tests

#### Solute and solid tracers

Two types of tracers were used for this study: (1) floating biodegradable wood chips, and (2) fluorescent dyes in the form of the conservative tracer uranine (Acid Yellow 73) and the nonconservative tracer rhodamine WT (Acid Red; Field et al. 1995; Leibundgut et al. 2009). Sampling of fluorescent dyes follows the principle of excitation of a water sample and measuring the ensuing emission intensity. The fluorescence of a dye tracer is pH-dependent, and not considered to be a factor in this environment where the pH is approximately 7. Solubility is 300 g/L for uranine compared to 3-20 g/L for rhodamine WT (Leibundgut et al. 2009). Excitation and emission maxima for uranine are 491 and 516 nm respectively, and 561 and 586 nm for rhodamine WT.

The sorption behaviour of tracers is particularly important with regard to the quantitative analysis. In general, the higher the solubility in water, the lower the sorption capacity of the substance. Rhodamine WT shows a strong sorption behaviour, while uranine has a very low sorption tendency (Field 2002) which applies to the given environment.

Given the uncertainty of SGD locations in the sea, the suitability of floating wood chips as a natural and biodegradable tracer was tested. The reasoning for using wood chips was to mitigate the problem of spatial integration (dispersion) related to solute tracers: there is no detection limit as the wood chips are measured by visual observation. Hence, a single detected floating wood chip may be sufficient to confirm the success of the tracer test. The ideal solid tracer would have a density between freshwater (1 kg/m^3^) and seawater (~1.035 kg/m^3^) in order to be neutrally buoyant within the freshwater underground river and conduits of the aquifer before being discharged into the sea where it would float up to the sea surface and thereby be visually detectable.

The buoyancy of five different types of wood was tested in a stirring freshwater tank over the period of 10 days, ranging in density from 660 to 960 kg/m^3^ (TRADA 2018). The chips tested were of the size 4 × 40 × 50 mm. The best performance in terms of neutral buoyancy in freshwater was achieved by Keruing (*Dipterocarpus spp.*). When salinity was increased, the buoyancy increased. A total of 250-L of Keruing chips were manually produced, soaked in freshwater and packed in 10 × 30 L caving bags. The concentration of wood chips >2 mm grain size was counted as >1,000 parts per 50 ml—ergo, the total amount of chips (>2 mm) for the volume of 250 l was estimated at 5.71 million.

The geophysical analysis of the submarine sinkholes considered as potential SGD locations, show both the presence and absence of a sediment infill (O’Connell et al. 2018). Such sediments might play a role in blocking or attenuating the solid wood, which needs to be considered in the interpretation of results.

#### Injections

The two fluorescent dyes and wood chips were injected at three different sites on 14 April 2018: two injection sites located within the cave Poll Gonzo with the entrance 7.5 km south of Bell Harbour Bay and the openly accessible sinkhole Deelin Pot 5.9 km south of Bell Harbour Bay.

Access to Poll Gonzo required a team of six cavers to abseil three pitches of ~20 m each on fixed ropes. The uranine was dissolved in-situ within a 100-L inflatable swimming pool and released into the underground stream upstream of a waterfall at ~70 masl and 56 m below ground level (Table 2, site 1a).

**Table 2:** Injection site coordinates, elevation, tracer mass and type, injection time on 14 April 2018 and flushing volume

Injection site	Coordinates	Elevation [masl]	Tracer and mass	Injection time	Flushing volume
Site 1a Poll Gonzo waterfall	53.055°; −9.073°	~70	Uranine, 25 kg	13:00	~30 L/s
Site 1b Poll Gonzo bottom sump	53.055°; −9.073°	~30	Wood chips, 250 L	12:00	~30 L/s
Site 2 Deelin Pot	53.070°; −9.080°	~84	Rhodamine WT, 25 kg	17:00	2.7 m^3^

The 250 L of wood chips were released at the bottom of the cave at ~31 masl at the local water table, where three experienced speleologists abseiled down another pitch with difficult access (Table 2, site 1b). Rhodamine WT was injected into the bottom of Deelin Pot 1.67 km north-northwest of Poll Gonzo, where a 1 m^3^ carbon-fibre tank with a release valve at its bottom was installed to mix the dye in-situ (Table 2, site 2). The total volume of water used for dilution and flushing was 2.7 m^3^.

#### Monitoring of solute and solid tracers

Sampling for the tracers at potential outlets was conducted at terrestrial locations, as well as offshore along transects to account for the numerous potential SGD locations in Galway Bay. The terrestrial outlets were sampled using a fluorometer and/or activated charcoal kept in place for the period of observation. Detection of fluorescent tracers was done qualitatively using permeable nylon bags of activated charcoal pellets (technical grade, AppliChem Panreac). Each bag was filled with ~20-g pellets.

For off-shore sampling, GGUN-FL30 field fluorometers (Albillia Co., Switzerland) were used to (semi-) quantitatively analyse the fluorescence. GGUN-FL field fluorometers can measure three distinct dyes in parallel as well as turbidity at a sampling interval ≥10 s. Readings in mV are converted into ppb using a linear calibration to three tracer standards (1, 10 and 100 ppb) for each tracer made using local sea water and dye from the batch injected; hence, background fluorescence in the sea is broadly accounted for. The minimum detection limit for uranine in clear water is 0.02 ppb, and ≤0.2 ppb for rhodamine WT (Schnegg 2002). Visual monitoring for wood chips was conducted offshore from onboard of the vessel.

**Fig. 3:**
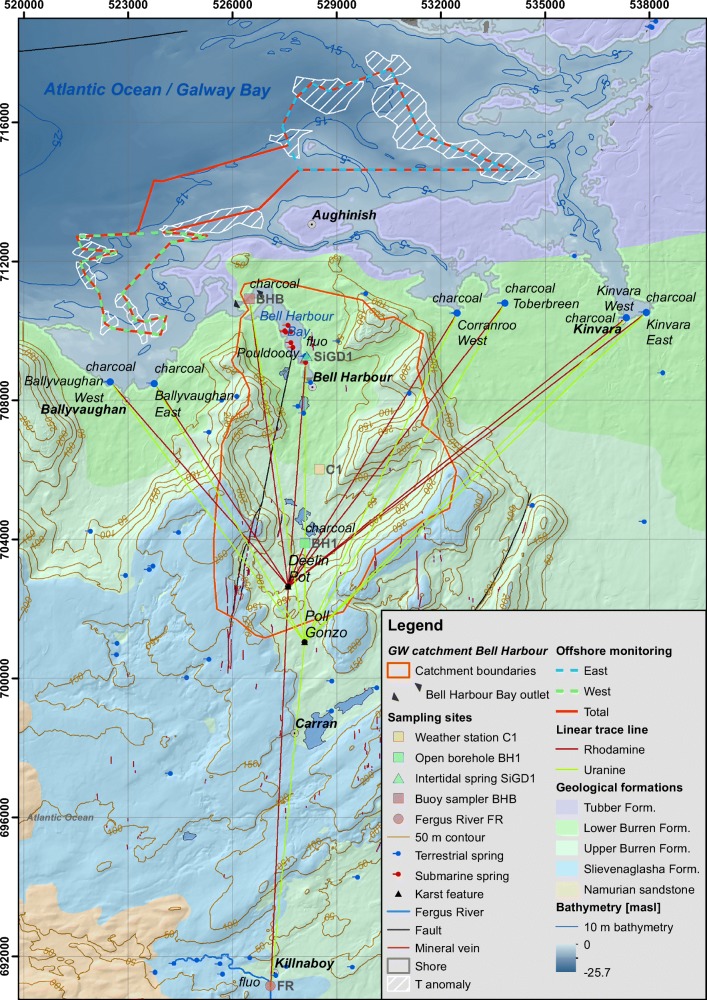
Overview of the tracer study including stationary onshore monitoring of intertidal springs, the outlet of Bell Harbour Bay (BHB), the Fergus River (FR) and the borehole (BH1) using field fluorometers and/or activated charcoal samplers, as well as mobile offshore monitoring transects across areas of temperature anomalies using a field fluorometer and CTD sensor

Stationary sampling was conducted at 10 different sites (Table 3; Fig. 3). A field fluorometer was installed at the Fergus River to assess the unlikely scenario of southwards transport. A second field fluorometer was installed at Pouldoody spring, along with a conductivity-temperature-depth (CTD) sensor to record the tidal fluctuation, temperature and conductivity as indicators for the occurrence of SiGD at Pouldoody spring.

**Table 3:** Location and instrumentation of the stationary terrestrial monitoring sites. Recording frequency refers to automated sampling while recording duration refers to the length of deployment of a charcoal sampler

Site ID	Observation site	Coordinates	Elevation [masl]	Distance to injection site [m]		Instrumentation	Recording frequency/ duration
				To Poll Gonzo	To Deelin Pot		
1	Fergus River at Crossard Bridge	52.966°; −9.085°	~23	9,392	10,878	Albillia GGUN-FL30	5 min; 14–25 Apr
2	Pouldoody spring	53.129°; −9.074°	−0.1	8,221	6,641	Albillia GGUN-FL30	5-15 min; 14–20 Apr
3	BH1, borehole	53.081°; −9.073°	15 to −170	2,848	1,339	Charcoal	14 to19 Apr, 19–25 Apr
4	Ballyvaughan West	53.122°; −9.158°	~0	9,343	7,796	Charcoal	14–19 Apr
5	Ballyvaughan East	53.121°; −9.139°	~0	8,607	6,994	Charcoal	14–19 Apr
6	Corranroo West	53.141°; −9.010°	~0	11,327	10,265	Charcoal	14–19 Apr, 19–25 Apr
7	Toberbreen	53.144°; −8.989°	~0	10,437	9,250	Charcoal	14–19 Apr, 19–25 Apr
8	Kinvara West	53.140°; −8.937°	~0	13,148	12,433	Charcoal	14–19 Apr, 19–25 Apr
9	Kinvara East	53.142°; −8.928°	~0	13,659	12,974	Charcoal	14–19 Apr, 19–25 Apr
10	BHB, outlet of Bell Harbour Bay	53.144°; −9.099°	~0	10,111	8,356	Charcoal	14–25 Apr

Qualitative sampling methods using activated charcoal bags varied by site. Within the open borehole (BH1), four bags were tied on a 200-m-long bottom-weighted rope hung in the borehole to sample depths ranging from 15 to −170 masl. The six known intertidal coastal springs were monitored with individual bags tied to concrete slabs at the intertidal outlets: Ballyvaughan West and East, Corranroo West, Toberbreen and Kinvara West and East. Furthermore, two charcoal bags were attached to a floating buoy anchored in the seabed in the centre of the outlet of Bell Harbour Bay (Fig. 1), sampling the top sea water column. The high tidal range largely empties Bell Harbour Bay during ebb tide, such that fluorescence may have been detected at that site. The charcoal bags were collected on 19 April and the replacement set collected on 25 April.

Mobile sampling of sea surface fluorescence and conductivity over transects was conducted over 21 h on four successive days from a vessel in the sea targeting the areas of temperature anomalies previously identified by remote sensing. Sampling focused on the central and western area, as the temperature anomalies were considered to be more explicit than those of the east, which may be due to freshwater dilutions from Kinvara Bay/surface water. Based on estimated travel times (Table 1), sampling started 36 h after injection and lasted for 4 days. The fluorometer was tied on ropes pulled by the vessel, while also cabled to the data logger and a computer on board. The ropes were weighted to make sure the fluorometer did not float, sampling between 0.03 and 5.77 m (average 1.01 m) below the sea surface. In addition, the speed of the vessel was very slow at 3–5 km/h to reduce perturbation and erroneous readings due to air bubbles from the propeller. A CTD sensor was attached close to the fluorometer to sample for depth, temperature, and conductivity as indicator for SGD. A hand-held global positioning system (GPS; GPSmap 60CSx, Garmin, KS, USA) was synchronised with the fluorometer and CTD sensor, recording the geographic position of the vessel in 3-s intervals.

#### Analysis of solute and solid tracer results

Charcoal bags were collected on 19 and 25 April 2018, 5 and 11 days after the injections, respectively. All sample bags were individually sealed and kept protected from sunlight in a closed container. The charcoal samples were analysed within days following collection. The Smart Solution was used for rhodamine WT consisting of 1-propanol, deionized water and 30% NH_4_OH in the ratio 5:3:2 (Smart and Simpson 2002). For uranine, the eluant was a mixture of 70% isopropyl alcohol, 30% high purity deionized water and 10 g/L sodium hydroxide. The analysis was carried out using a Cary Eclipse Fluorescence Spectrophotometer (Agilent Technologies, Santa Clara, CA, USA) based in the environmental laboratory of the GSI in Dublin, Ireland. The samples were analysed in sequence; hence, the elution time of a charcoal sampler ranged from at least one, to a maximum of 2 h.

In contrast to the rather clean continuous signal achievable with terrestrial stationary monitoring, sampling in the sea is impacted by perturbation through currents, drag of the boat, and air bubbles causing potentially erroneous readings. Hence, potentially erroneous readings were filtered following data acquisition. The filter compares the ratio of observed mV signals of given tracers with the ratio of mV signals of the same tracers as established during the calibration procedure using the values of the corresponding photodetectors which were L1 and L2 for the two tracers rhodamine and uranine, only the ratios of L1 and L2 were recorded in the raw files.

First, the ratio of measured mV signals for a tracer *T*_*n*_ of interest is established using the measured signals of L1 during calibration *L*1_cal_ [mV] and L2 during calibration *L*2_cal_ [mV]. The calibration ratio *r*_0_ is then given by Eq. (1),

1 $$ {r}_{0,{T}_n}=\frac{\ L{2}_{\mathrm{cal},{T}_n}-L{2}_{\mathrm{cal},W}\ }{L{1}_{\mathrm{cal},{T}_n}-L{1}_{\mathrm{cal},W}} $$

with the associated signal for pure water *W* [mV] and the tracer with *n* = 1 for uranine and *n* = 2 for rhodamine.

The result is a characteristic ratio for each of the three tracers that can be monitored, corrected by subtracting the background signal of water. The actual observed ratio between photodetector 1 *L*1_obs_ [mV] and photodetector 2 *L*2_obs_ [mV] at time step *t* is established to yield *r*_*t*_ following Eq. (2)

2 $$ {r}_t=\frac{\ L{2}_{\mathrm{obs}}-L{2}_{\mathrm{cal},W}\ }{L{1}_{\mathrm{obs}}-L{1}_{\mathrm{cal},W}} $$

again, corrected for the background value of water.

Ideally, ${r}_t={r}_{0,{T}_n}$ but this is never exactly the case. Since L2 is measured 100 ms after L1, the measurement is rejected if a large deviation of *r*_*t*_ from ${r}_{0,{T}_n}$ is observed, indicating the occurrence of air bubbles during the measurement of L1 or L2. Here, an observation was considered reliable if its *r*_*t*_ ranges between the lower and upper limit of the ratios of the two tracers considered, which are *r*_1_ = 0.035 for uranine and *r*_2_ = 2.597 for rhodamine. In addition, a threshold deviation of ±20% was defined as acceptable so that $0.8\times {r}_{0,{T}_1}<{r}_{t,{T}_n}<1.2\times {r}_{0,{T}_2}$.

Because of its nature, the method is called ‘quotient method’. This approach was considered to be very conservative in order to reliably define the validity of a single tracer reading following a transparent filtering sequence.

## Results

### Remote sensing

The calculated temperatures are displayed in Fig. 2a–d. The aim is to highlight the relative temperature difference as this is the parameter of interest for this analysis. The water surface of Galway Bay shows a relatively consistent temperature (except Fig. 2). Localised cold temperature anomalies were interpreted as potential influence of SGD, which change in position over the 4 months of the year shown in the images. This may be caused by the presence of multiple SGD locations, currents and different mixing dynamics in the sea over the year, and between years. Another aspect to be considered is water inflow from the shore: Fig. 2a shows two large temperature anomalies in the east, which are likely related to cool discharge from Kinvara Bay (Gill et al. 2013; Schubert et al. 2015) and surface-water inflow. Table 4 gives an overview about the mean and standard deviations of surface temperatures of eastern Galway Bay (extent of the map in Fig. 2) and identified temperature anomalies, as well as their differences. These temperature anomalies were considered to be potentially related to SGD. Temperature anomalies differ the most between the January 2017 and November 2016 images; therefore, these areas were considered as most relevant for sampling.

**Table 4:** Mean and standard deviation (*SD*) of temperatures (*T*) in the eastern part of Galway Bay and within temperature anomaly polygons in °C, as well as their difference, ordered by month of the year

Image date	*T* Galway Bay [°C]		*T* anomalies [°C]		Difference [°C]	
	Mean	SD	Mean	SD	Mean	SD
2 January 2017	7.5	0.89	5.0	0.37	2.5	0.52
8 April 2017	12.6	0.69	12.0	0.20	0.6	0.49
11 July 2013	20.7	1.10	19.2	0.22	1.50	0.88
24 November 2016	10.7	0.80	8.80	0.23	1.9	0.57

### Artificial tracer tests

Figure 3 outlines the conceived pathways between the two injection sites Deelin Pot and Poll Gonzo and potential SGD outlets in Galway Bay, as well as the stationary terrestrial monitoring sites at intertidal springs, a borehole and the Fergus River using activated charcoal samplers or field fluorometers. The linear distances between injection sites and sampling locations are in Table 3.

Based on the temperature anomalies identified in section ‘Remote sensing’, transects were drawn that were used as reference to navigate the vessel iteratively throughout the areas of potential SGD. Since the temperature anomalies in the western part were more pronounced, sampling focussed in these areas by defining a western monitoring transect.

#### Stationary sampling (fluorometers and charcoal bags)

**Fig. 4:**
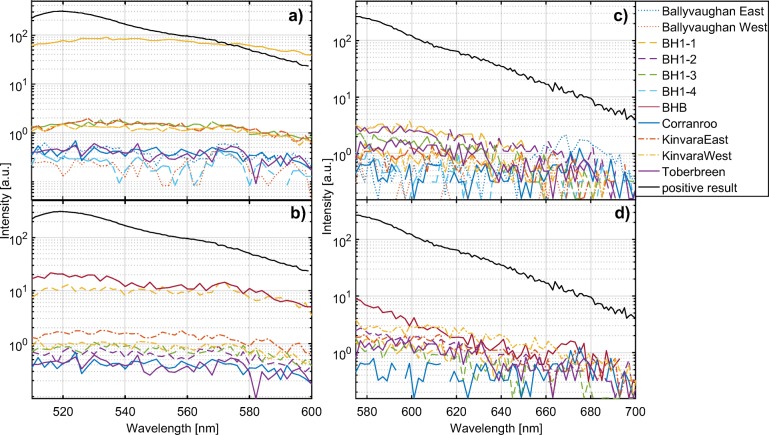
Emission spectra of activated charcoal samples for uranine collected on **a** 19 April and **b** 25 April and rhodamine collected on **c** 19 April and **d** 25 April

None of the emission spectra of the eluant from charcoal samples collected on 19 and 25 April show emission peaks of 516 nm for uranine (Fig. 4a,b), nor an emission peak of 586 nm for rhodamine (Fig. 4c,d) samples. All charcoal samples are interpreted to be free of any of the two dyes.

**Fig. 5:**
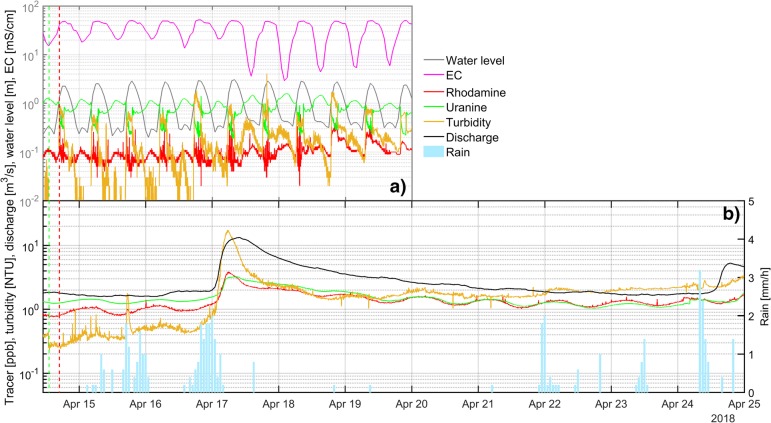
**a** Concentration of uranine and rhodamine in ppb, turbidity in NTU and water level in m observed at the intertidal spring Pouldoody between 14 April and 20 April; and **b** readings of uranine and rhodamine in ppb, turbidity in NTU and discharge in m^3^/s observed at the Fergus River between 14 April and 25 April, as well as hourly rainfall measured at C1. The green- and red-dashed vertical lines indicate injection times of uranine and rhodamine, respectively

The time series fluorometry (Fig. 5a) shows the results obtained from Pouldoody spring, along with water level fluctuations measured at the monitoring site. The obvious twice-diurnal water-level fluctuation is a function of the tidal cycles. Further, the fluctuation of conductivity indicates active discharge of the spring with notable minimum values of up to 2.9 mS/cm during low water levels starting on 17 April. Groundwater discharge is at maximum when the observed EC is at its minimum as a result of the low water level (ebb tide). At such times, the recorded concentrations of rhodamine and uranine peak <0.3 ppb and slightly above 1 ppb, respectively. The impact of the tidal fluctuation on the readings of both dyes is believed to be the result of changing chemistry and composition of the water at the site, probably dissolved organic matter (DOM). As a consequence of these low concentrations, the results are interpreted as negative.

Figure 5b shows the result of the field fluorometer installed at the Fergus River to the south of the tracer injection points. The river flow exhibits a clear response to the two preceding rainfall events of 11.6 mm (15 April 3:00 to 16 April 1:00) and 14 mm (16 April 14:00 to 17 April 4:00). As a result, discharge increases from 1.6 to 12.8 m^3^/s along with concurrent increases in turbidity. Uranine and rhodamine show both a correlation with turbidity peaking at 2 and 3 ppb respectively. Correlation between fluorescent dyes and turbidity within this range is common (Schnegg 2002). In addition, measured fluorescence exhibits a negative correlation with the temperature in the stream; hence, altogether, the observed dye concentrations are interpreted as a mixture of natural background and/or anthropogenic sources.

#### Mobile sampling

**Fig. 6:**
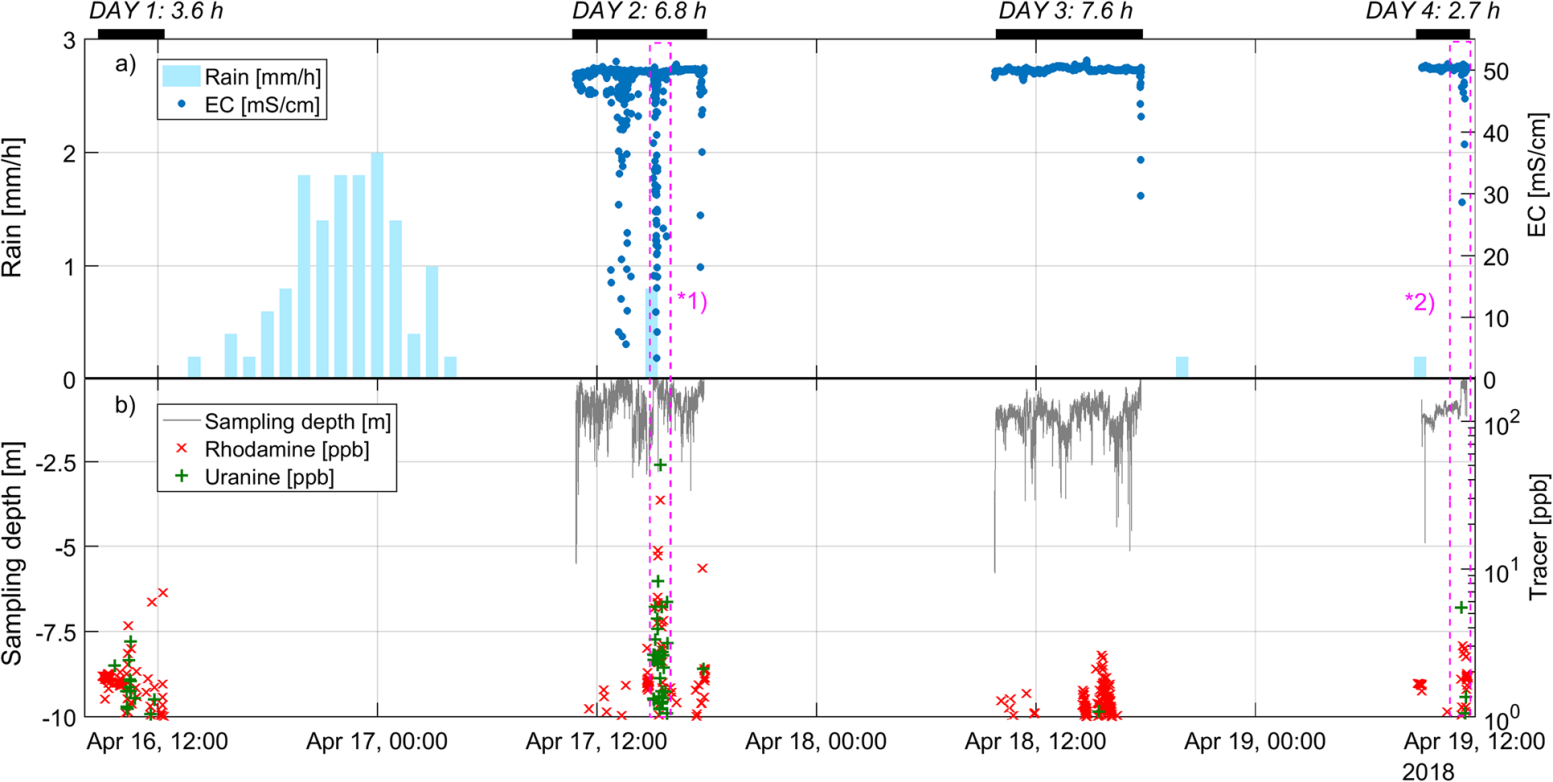
**a** Hourly rainfall and electrical conductivity (EC) in mS/cm recorded offshore; **b** Filtered records of concentrations of rhodamine and uranine in ppb plotted with the depth of the CTD sensor and field fluorometer in m below surface. The black bars on the top show the duration of sampling for each day. The pink-dashed rectangles *1 and *2 highlight short periods of low conductivity and high tracer readings (day 2: 15:05–15:20; day 4: 11:15–11:28)

Figure 6a,b shows the concentrations of uranine and rhodamine for the filtered data in semi-log scale along with measured conductivity in the sea, depth of sampling and hourly rainfall. Conductivity records are missing for day 1 (16 April). Monitoring was limited to the eastern transect on day 1 due to very harsh weather conditions and a red weather warning for Galway Bay issued by the Irish meteorological service MetEireann. During days 2–3, sampling focussed to the centre and the western transect, for the reasons previously discussed, and that more elevated tracer readings were noticed in real-time in the centre and the west on day 2.

The raw data show 4,998 readings with maximum values for rhodamine and uranine reaching 208.6, 457.8 ppb respectively (Fig. 6b). The quotient method filtered 12.7% potentially erroneous readings, leaving 4,365 readings or 87.3% to be considered (Fig. 6b). As a result, the maximum concentrations of rhodamine and uranine considered reliable are 29.3 and 50.7 ppb respectively.

Both maximum dye concentrations were recorded on sampling day 2 (17 April) which correlate with the lowest conductivity <5 mS/cm values also recorded on day 2. Periods of low conductivity and high tracer readings are highlighted in pink rectangular dashed boxes (day 2: 15:05–15:20; day 4: 11:15–11:28). Importantly, the fluorometer and CTD sensor were very shallow during these two periods (0.18, 0.23 m). The average sampling depths on days 1, 2 and 3 are 0.81, 1.20 and 0.96 m, respectively (average of 1.01 m). Low conductivity levels may be linked to the two rainfall events between 15 April and 17 April with a total of 22.6 mm. It is hypothesised that the rapid recharge increased SGD with the resultant lowering of conductivity in the sea at these locations. In comparison, the conductivity on the 18 and 19 of April, during which there was no rainfall, are much more homogeneous and relatively constant. It is assumed that without rainfall, the rate of SGD is lower. No correlation between conductivity and tracer concentration occurred on day 3 with the conductivity readings remaining high at 50 mS/cm, with exceptions.

**Fig. 7:**
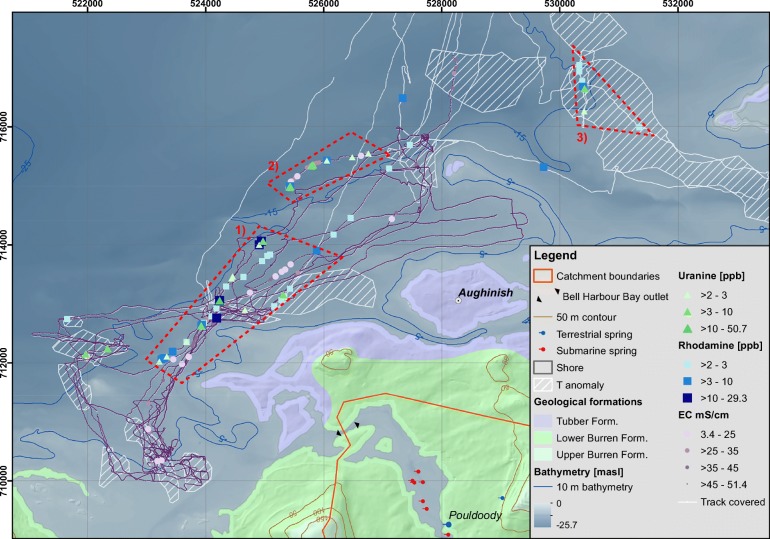
Results of offshore sampling between 16 and 19 April 2018: measured and corrected tracer concentrations of rhodamine and uranine (>2 ppb) along with measured electrical conductivity (EC). The track of the vessel is plotted but covered by conductivity readings where available. Previously identified temperature anomalies are plotted. Three areas, shown in hashed boxes nos. 1, 2 and 3, show concentrated tracer readings. In areas 1 and 2, tracer readings correlate very well with conductivity readings

The fact that high tracer readings correlate in time and space with low EC readings suggest consistency of the method. Overall, it was assumed that a large amount of dilution weakens and spatially integrates the signal, and therefore a clear correlation cannot be expected. In order to evaluate the spatial pattern of recorded values, the georeferenced and filtered ppb readings for rhodamine and uranine were mapped, along with monitored conductivity of the sea water and the total track recorded by the GPS (Fig. 7). Both dyes are plotted for values >2 ppb in order to not overload the map with low (and presumably not meaningful) concentrations.

In general, tracer was recovered in a relatively large geographical area, mostly in three clusters: cluster 1 in the west, cluster 2 in the centre and cluster 3 in the east. In all clusters, both dyes were recovered. Altogether, a spatial correlation between dye readings and conductivity occurred. Cluster 1 contains rhodamine and uranine readings of days 2–4. This cluster contains the maximum observations for rhodamine and uranine reaching 29.3 and 50.7 ppb respectively, associated with low conductivity readings down to 3.4 mS/cm on day 2 (Fig. 6, *1) corresponding to travel times of ~160 and 165 m/h for rhodamine and uranine. Also, Cluster 2 relates mainly to the highly correlated low conductivity and high tracer readings observed on Day 2 (Fig. 6, *1) with associated travel times of ~>181 and 194 m/h for rhodamine and uranine. These two areas are considered as SGD ‘hot spots’.

The longest distance between injection sites and sampling locations occurred in cluster 3, where uranine was sampled 15.8 km away from Poll Gonzo and rhodamine 14. 6 km away from Deelin Pot, which correspond to travel times of ~331 and 339 m/h. No wood chips were observed during offshore monitoring. During the first 3 days of sampling, the sea was very rough due to strong winds with maximum average hourly wind speeds of 8–11 m/s. These conditions made the work onboard difficult and monitoring of wood chips on the sea surface challenging. On day 4, the sea became very calm, but no wood chips were seen or sampled; therefore, the result of the tracer test using wood chips is considered as inconclusive/negative.

## Discussion

Potential SGD areas were identified using Landsat 8 OLI images. The resulting temperature anomaly maps show different areas of potential SGD in time. The reason for the different spatiotemporal pattern is believed to be the multiple outlets and different mixing patterns in the sea, modifying the signal of SGD dispersion in the sea and associated temperature patterns (Breier et al. 2005). Accordingly, the use of different temperature maps proved to be of use in designing the offshore sampling strategy.

Sampling of fluorescence in the sea using a field fluorometer is a challenge. Different factors, particularly the weather conditions, created limitations in sampling. Potentially erroneous readings were observed, most likely related to air bubbles causing perturbation problems. Potentially erroneous data were filtered out using a new developed quotient method, leaving 87% of the observation points for analysis. The quotient method is considered to be conservative, with positive results considered to be valid. In the future such problems could be mitigated by using a better, bubble-free location of the fluorometer, e.g. under the vessel. Similarly, this applies to the conductivity readings, which may be impacted by air bubbles; however, in this study, the conductivity readings did not show any evidence of erroneous readings. The majority of readings were consistently close to 50 mS/cm, with lower readings occurring systematically in some locations, and also correlating with elevated tracer readings on days 2 and 4. The combination of consistent readings and correlation with tracer readings in time and space are interpreted as a strong indicator for the validity of the tracer and conductivity time series.

None of the charcoal samples placed at the intertidal springs along the shore, the borehole (BH1) and the outlet of Bell Harbour Bay (BHB) showed evidence of adsorbed dye. Hence, the analyses of the charcoal samples were interpreted as negative, the results being consistent with previous tracer studies carried out in the uplands in the area (Bunce and Drew 2017). Factors such as the period of elution and the composition of the sampled water, including the presence of organics, generally impact on the intensity of the analysed spectra (Smart and Simpson 2002; Wernli 2011); hence, “water tracing in karst is an inexact science” (Bunce and Drew 2017). A hydraulic connection between the two injection sites and the sampling sites for the period of the tracer test could neither be confirmed nor rejected.

Submarine groundwater discharge (SGD) in Galway Bay is likely to occur via sinkholes that are filled by sediments. Such sediment infill may have trapped the solid wood chips, providing one explanation for the negative trace using wood chips. Alternatively, the wood chips could be trapped in conduits or cavities en route, or, they may have been discharged at sea, but they could not be observed due to the particularly rough weather conditions over the first 3 days. Further, the total parts of visible wood chips (>2 mm) is 5.17 million, which is insignificantly small compared to the total parts of fluorescent dyes injected (25 kg of both uranine and rhodamine) with respect to their detection levels <1 ppb. Given these circumstances, the chance of detecting wood chips visually during the 4 days of sampling can be considered much lower than detecting the fluorescent dyes using the field fluorometer.

Besides the negative results, the two fluorescent dyes were successfully sampled in the sea. Single observation-based travel times range between 100 and 354 m/h. These times are in the order of the estimated travel times previously conducted in the area (Table 1). The depth of the CTD sensor and fluorometer ranged from 0.03 to 5.8 m below the surface during the sea transects, largely impacted by the speed of the vessel. It is notable that the highest correlation between tracer readings and low conductivity levels was recorded at relatively shallow depths (0.18–0.23 m). This indicates that freshwater SGD floats as a layer on the sea surface; therefore, for a future offshore monitoring campaign, it is recommended to fix the fluorometer and CTD sensors at shallow depths of <0.25 m or less below the sea surface.

The sampled tracer in the sea only partly overlaps with the areas previously identified on the temperature anomaly maps. This observation highlights the complexity of SGD studies with regard to the existence of potentially multiple outlets and subsequent mixing and dispersion dynamics in the sea. At the same time, variables that influence the dynamics of SGD such as tides, waves and currents (Gill et al. 2013; Parra et al. 2016) were not considered in the design of the study and interpretation of the results, but their understanding may improve the understanding of the SGD regimes.

Given the positive results in the sea and negative results along the shore, the study suggests that the two dyes may have bypassed the sampled intertidal springs, potentially at deeper levels. Such flow paths will have developed during periods of lower sea levels tied to global eustatic sea level variations (Shennan et al. 2018). The occurrence of significant deep groundwater circulation including a bypassing effect of intertidal springs had been proposed based on long-term monitoring, quantification of SiGD, water balance calculations and numerical modelling (Bunce and Drew 2017; Schuler et al. 2018). Such deep flow is hypothesised to be linked to preferential flow paths along north/north–north–west-trending Variscan veins (Moore and Walsh 2013); hence, this study supports the relevance of these structural features with regard to regional groundwater flow.

## Conclusion

Submarine groundwater discharge (SGD) is recognized as an important pathway for contaminant transport into the coastal environment; hence, it is of relevance in the context of coastal karst catchments. A good understanding of SGD dynamics linked to the drainage of coastal karst aquifer is therefore necessary.

This study combines methods to: (1) locate potential areas of SGD using first remote sensing to; (2) apply a set of different artificial tracers, including fluorescent dyes as well as floating wood chips, and (3) evaluate hydraulic connections between terrestrial injection points and offshore submarine discharge locations. The method was successfully applied in the study area of the coastal aquifer of Bell Harbour in western Ireland.

None of the wood chips were recovered from the sea. Different factors may have prevented a positive result, including the potential sediment infill of submarine springs, trapping these solid particles.

Both of the fluorescent dyes were recovered in the sea, and up to 15.8 km away from an injection point. Offshore sampling was conducted in transects in the sea over four successive days onboard a vessel. The estimated travel times are in the order 160–339 m/h or less. Fluorescence peaks correlate with lower conductivity values in the sea, indeed indicating the discharge of fresh/brackish groundwater. Two main areas of SGD were identified, whereas the spatial pattern of SGD is believed to reflect multiple-point SGD locations. It is hypothesised that the main outlets of the catchment are located in clusters 1 and 2; however, additional studies are needed to confirm this conclusion.

Hydraulic connections were established between two structural karst features of the upland of the Burren Plateau and Galway Bay. Both structural features include mineral veins, supporting the hypothesized relevance of Variscan veins with regard to regional groundwater flow control (Moore and Walsh 2013; J. Walsh et al., University College Dublin, unpublished paper, 2019).
